# Effects of mealtime assistance in the nutritional rehabilitation of eating disorders

**DOI:** 10.1007/s40519-023-01605-9

**Published:** 2023-09-09

**Authors:** Doriana Lacalaprice, Edoardo Mocini, Francesco Frigerio, Marianna Minnetti, Claudia Piciocchi, Lorenzo Maria Donini, Eleonora Poggiogalle

**Affiliations:** Department of Experimental Medicine, Sapienza University of Rome, Rome, Italy

**Keywords:** Eating disorders, Eating psychopathology, Eating disorders treatments, Nutritional therapy, Mealtime assistance

## Abstract

**Purpose:**

The aim of the study was to examine the effects of meal supervision, provided by health professionals, volunteers or family members, on anthropometric, nutritional, psychological, and behavioural outcomes in patients with eating disorders (EDs).

**Methods:**

The present systematic review was performed according to the Preferred Reporting Items for Systematic Reviews and Meta-Analyses (PRISMA) statement. The last search was conducted in three databases (PubMed, Scopus, and the Cochrane library). Inclusion criteria considered paediatric and adult patients suffering from EDs, regardless of ethnicity, and treated in different therapeutic settings. The quality of the studies was evaluated using the Newcastle Ottawa Scale (NOS) adapted for cross-sectional studies and Version 2 of the Cochrane risk-of-bias assessment tool for randomised trials.

**Results:**

3282 articles were retrieved, out of which only 6 met the eligibility criteria. A marked heterogeneity in definitions and approaches to supervised mealtime was observed. This variability emerged in the methodologies used in the supervised meal, and in the reference values for the outcome measures that were used, such as the analysis of different parameters.

Based on these observations, mealtime assistance provided to patients with EDs shows an overall positive effect on eating behaviour and dysfunctional attitudes. Future research should be prompted to provide a thorough definition of a structured procedure for meal assistance to be potentially and systematically included in the nutritional rehabilitation protocols for patients with EDs.

**Level of evidence:**

Level IV systematic reviews of uncontrolled trials.

## Introduction

Eating Disorders (EDs) are a large group of disabling pathologies, classified among psychiatric disorders, which compromise the physical health and cognitive, emotional and social functioning of the individual [1]. They are characterised by alterations of eating habits and excessive concerns for body weight and body shape [2]. Nutritional rehabilitation interventions represent an essential pillar of the treatment for patients with EDs [3].

It is important to ensure early interventions, through age-specific nutritional assessment, diagnosis and treatment, particularly in relation to the significant reduction in the age of onset of EDs and the related clinical consequences associated with early onset, such as the occurrence of malnutrition before full pubertal development [1, 4]. Treatments for adult patients, as well as for patients who are in the pubertal or prepubertal stages, require a personalised approach of psycho-nutritional rehabilitation, starting from the actual abilities of the patient and relating to the desired outcomes [5–7].

Nutritional therapies are aimed at either reducing or eliminating inappropriate compensatory behaviours, and interrupting purging episodes—if present—to restore normal eating behaviour and adequate nutritional status. The clinical conditions to be addressed encompass the restrictive behaviours associated with Anorexia Nervosa (AN), the loss of control during a bulimic crisis in patients with Bulimia Nervosa (BN) or Binge Eating Disorder (BED), the compensatory behaviours, such as self-induced vomiting, the abuse of laxatives and diuretics, the abuse of alcohol and drugs, and the major psychological psychiatric comorbidities, often present at the time of diagnosis and associated with a poor adherence and reduced compliance to treatments [8].

Several treatment options are available to treat EDs, ranging from inpatient hospitalisation to outpatient services and various therapeutical strategies [9]. Mealtimes can be a challenging experience, aimed at restoring eating behaviours [10, 11], promoting adequate calorie consumption, fostering appropriate weight gain [12] and preventing hospital admission [10].

Health-care professionals play a prominent role in helping patients to restore normal eating patterns during mealtimes [10, 13, 14]. The approach of the professional figures involved is structured in relation to the severity of the disorder and the degree of impairment of the clinical situation [15]. In the context of clinical rehabilitation for EDs, the nutritional intervention can be applied after the nutritional status evaluation relying on clinical and nutritional histories, the food counselling, the meal planning, the assistance during meals, the psycho-nutritional education, and the periodic measurement of anthropometric parameters and circulating biomarkers of the nutritional status. Regardless of weight restoration, eating behaviour is a strong predictor of clinical outcomes [16, 17]. Nonetheless, weight restoration is not fully associated neither with diagnostic recovery, nor with the risk of relapse [18, 19].

The assisted meal is one of the nutritional rehabilitation treatments suitable for patients suffering from EDs. The adherence to specific procedures during meals helps patients dealing with emotional states, as well addressing concerns related to the body appearance and physical sensations (e.g., the feeling of premature fullness, abdominal swelling) and food rituals (e.g., cutting food into small pieces, eating slowly). Although widely acknowledged as an important therapeutic activity, there is still a notable lack of evidence exploring and reporting cardinal aspects of meal structure and activities performed during mealtime assistance in patients with EDs [20]. The advantages of the clinical practice of assisted meals are mainly the possibility of restoring nutritional status and avoiding the need of intensive and invasive feeding methods.

This review focuses on the assisted meal practice in an integrated therapeutic strategy for patients with EDs. There are no standardised guidelines concerning the structure of the assisted meal and it is not thoroughly clear which protocols/strategies are most likely to be optimal, the procedures for mealtime management within different clinical settings and the clinicians’ experience [21]. In clinical practice, the assisted meal treatment has been carried out according to the empirical experience of health care professionals [22]. The development of a potential protocol on the structure and the procedures of the assisted meal could improve patient support to achieve the normalisation of eating behaviour, dietary habits, and body weight restoration.

## Aim

This systematic review (SR) summarises the effects of mealtime assistance on nutritional, anthropometric, psychological and behavioural outcomes in patients with EDs, as provided by either volunteers, caregivers, nurses, dietitians, or other specifically clinical trained staff. Secondary objectives of the research are aimed at evaluating the effects of mealtime assistance on general health status and medical complication with a particular focus on vital signs of patients with EDs. The main purpose of the research is, therefore, to extract and synthesise extant evidence concerning assisted meals, with the aim of identifying current procedures for mealtime management.

## Materials and methods

### Literature search

Last search was conducted until March 31st 2023, in three different databases: PubMed, Scopus and the Cochrane library. Additional articles of potential relevance were also manually searched both through the reference articles included or excluded in the review and according to the suggestion of the other authors of the study.

The search was conducted based on the following search terms: (“*eating disorders*”) AND (“*meal supervision*” OR “*mealtime assistance*” OR “*meal therapy*” OR “*mealtime intervention*” OR “*nutritional rehabilitation*”). The searches from the three independent databases were combined and duplicates were removed to create a master file used for titles and abstracts screening. In addition, neither language restrictions nor time limits have been applied in the selection of studies.

### Eligibility criteria

The PICOs format (Participant–Intervention–Comparator–Outcomes–Study design) was used to develop the criteria for study inclusion (Table 1) [23]. The criteria for inclusion of articles in the review considered cohorts of paediatric and adult patients of any ethnic origin, suffering from eating disorders and receiving mealtime assistance by healthcare professionals (e.g., nurses, dietitians, psychologists), family members or volunteers. Study designs including multiple interventions with mealtime assistance reported as an independent intervention were eligible for inclusion.

**Table 1:** Summary of inclusion and exclusion criteria applied during the evaluation of studies for the systematic review

Population	Adult and paediatric patients with eating disorders, of any ethnicity
Intervention	Mealtime assistance program
Comparator	Standard/usual care
Outcome	Nutritional status, anthropometric parameters, psychological effects, eating behaviour, general health and medical complications
Study design	No criteria applied

### Study selection

Studies were selected using the Preferred Reporting Items for Systematic Reviews and Meta-Analyses (PRISMA) statement process of identification, screening and assessment of eligibility [24]. The selection of the studies was performed in a three-step process involving the evaluation of (1) titles, (2) abstracts and (3) full-texts. If the title and/or abstracts were relevant, the full article was then obtained to determine eligibility. Two investigators (LD and EM) independently screened for eligibility at each step. Those that did not meet inclusion criteria were excluded. Any inconsistencies were resolved through consensus. In the case of a discrepancy between investigators, a third investigator from the coordinating team resolved each case through a discussion with the reviewers until a consensus was reached and articles were either excluded or moved to the next stage.

## Results

### Search results

**Fig. 1:**
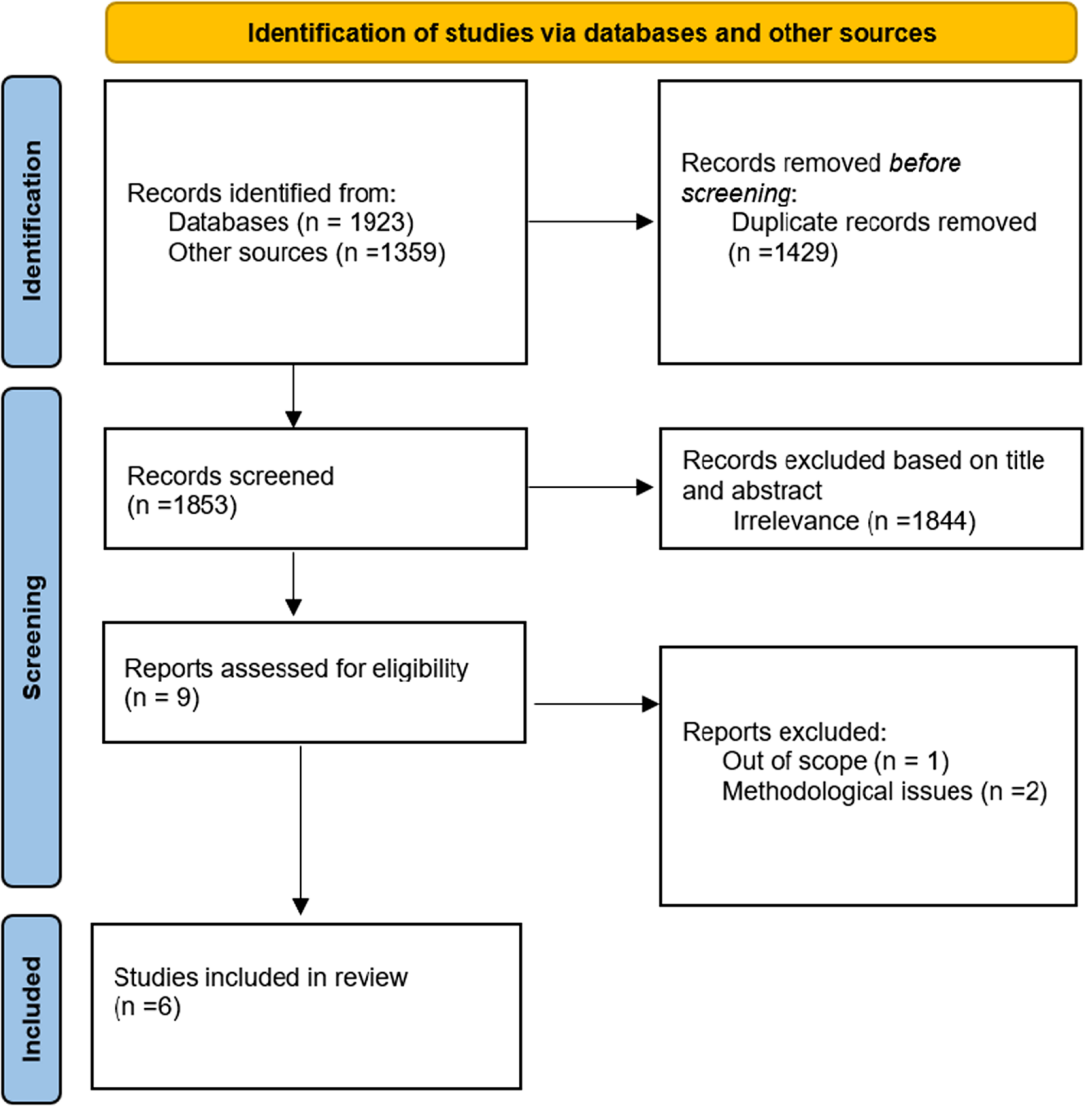
PRISMA 2020 statement (Page et al. 2021 [24])

The study selection process is presented in Fig. 1. The electronic search retrieved 3282 references. After removing duplicate references, a total of 1853 titles and abstracts were screened for eligibility with 1844 excluded at the first pass stage. The full text of the remaining nine articles was reviewed. In sum, a total of nine references were selected for full text evaluation and only six articles were included in the SR. The reason for full text manuscripts being excluded was being out of scope and methodological issues due to the absence of statistical analysis.

### Study characteristics

The main characteristics of the six articles selected are summarized in Tables 2 and 3. All were published between 2006 [25] and 2021 [26] and the total number of participants included was 314 patients with a sample size ranging from 12 [25] to 103 [27] participants. The majority of patients were women and the mean age of the study participants ranged from 14.9 years [28] to 33 years [26]. Studies were conducted in different continents, including three studies in the USA [12, 27, 28], one in the UK [26], one in Canada [29] and one in Argentina [25]. All full-text studies included were in English, except one, available only in Spanish [25].

**Table 2:** General characteristics of the studies

	Country	Sample size (*n*)	Gender (*n*)		Age (years ± SD)	Study design
			M	F		
Bravender et al. (2017) [28]	USA	82	2	21	9–21	Observational study
Coutrier et al. (2009) [29]	Canada	21	2	19	15.1 ± 1.9	Retrospective chart study
Gardner et al. (2021) [26]	UK	44	0	44	19–68	Prospective cohort study
Herscovici et al. (2006) [25]	Argentina	12	0	12	17.5 ± 2.1	Randomised pilot study
Kells et al. (2013) [12]	USA	52	2	50	15.9 ± 2.5	Retrospective chart study
Kells et al. (2017) [27]	USA	103	8	95	14.8 ± 2.3	Retrospective chart study

M: Males; F: Females

**Table 3:** Characteristics of meal assistance interventions

	Kells et al. (2017) [27]	Kells et al. (2013) [12]	Herscovici et al. (2006) [25]	Gardner et al. (2021) [26]	Coutrier et al. (2009) [29]	Bravender et al. (2017) [28]
Setting and ED diagnoses	Hospitalized patients with a diagnosis of ED NOS, AN (both either restrictive or purging types) or “pre-diagnosis”. Diagnostic criteria not mentioned	Hospitalised patients with REDs. Specific diagnosis of the disorder and diagnostic criteria not mentioned	Outpatients with a diagnosis of AN according to DSM-IV or the *Great Ormond Street* diagnostic criteria for early onset AN	Patients with AN in inpatient unit. Subtype of AN and diagnostic criteria not mentioned	Hospitalised patients with a diagnosis of AN (restricting or binge/purge type) Diagnostic criteria not mentioned	Hospitalised patients with AN or BN. Subtype of AN non specified
Definition of the mealtime procedure	“Meal supervision may be a supportive intervention to aid in meal completion and weight gain”	“Meal supervision allows for addressing disordered behaviours as they occur and encouraging food consumption”	“Mealtime assistance is treatment with the role of emphasising a collaborative environment and encourage to gradually increase food intake”	“Mealtimes are a mainstay of treatment for EDs”	“Meal assistance is a therapy to normalize the eating behaviour of patients with EDs”	“The assisted meal is part of a hospital medical stabilization program in patients with EDs and plays an important role in providing the intake of the whole meal by the patients”
Meal assistance procedures	The staff was at the patient bedside for the duration of the meal, conversing with patient per individual patient preferences, avoiding topics of weight, body image, and calories. Patients were allowed to play music or watch TV during mealtimes. Staff provided reminders of time remaining for meal and encouragement to consume food on tray when necessary	Clinical staff as active and supportive observers during mealtime	Food was brought only for the patient, and the parents were invited to intervene only if the patient did not achieve the tasks, previously agreed during the motivational session	Food preparation, monitoring food intake, supporting and supervising patients in a positive and relaxed atmosphere	The staff member sat with the patient, or group of patients, throughout the meal or snack, providing empathy and understanding of the patient’s struggle, while encouraging the consumption. Distraction by talking about other issues, or by playing interactive games was encouraged	Patients sat within view of the nurse’s station in the doorway of their hospital room to eat or have a constant attendant with them to monitor their meals, whose calorie content is estimated by a dietitian
Staff members involved in meal management	Nursing staff	Health care professionals	Therapist, family members	Nurses, nursing assistants, dietitians, dietetic assistant, OTA, Physicians, psychologists	Dietitian, social worker, psychologist, psychiatrist	Nursing staff, dietitians (meal-planning)
Outcome	No significant differences in length of stay (*p* = 0.27), weight gain per day (*p* = 0.63), serum electrolytes, or vital signs (low heart rate, CI *p* = 0.73), average percent of days met “goal” weight (*p* = 0.78) for those who received meal supervision from admission	Supervised patients exceeded the weight gain goal of 0.2 kg/day (mean gain of 0.35 kg/day vs. 0.33 kg/day in those who did not receive meal supervision, *p* = 0.52) and reported fewer episodes of overnight bradycardia (15% vs. 40% than patients not receiving meal supervision, *p* = 0.20) Average of hospital stay was 2.5 longer in supervised group, even if not statistically significance (8.4 days vs. 5.9 in non-supervised group, *p* = 0.66)	Assisted meals do not have a significant effect on weight recovery (*p* = 0.39). The menstrual function was normalized in 5/6 patients receiving meal assistance and for 2/5 of the control group, though any statistical significance was not displayed.	Mealtime assistance reduced the average number of ED behaviours per patient in the dining room by 35% and reduced distress and anxiety for patients and staff The average length of mealtimes across a week reduced from 53 to 43 min Statistical significance was not available	The need of NG feeding was reduced (67% versus 11%, *p* < 0.02). No differences observed in length of stay (*p* < 0.39), change in body weight (*p* < 0.17), or readmission rates (*p* < 0.66). Rate of weight gain (kg/week) showed a higher rate in those receiving meal support therapy (*p* < 0.09)	Parents refer benefit in having a respite from supervision of meals at home on a five-point Likert scales ranging from 1 = *very unhelpful* to 5 = *very helpful* (4.34 points vs. 2.82 points, *p* = 0.001) Mean BMI increased during the hospital stay in patients under direct meal supervision, (15.3 kg/m^2^ at admission to 16.0 kg/m^2^ at discharge, *p* = 0.001; no control group available

AN: Anorexia Nervosa; BN: Bulimia Nervosa; CI: Confidence Interval; ED: Eating Disorder; EDNOS: Eating Disorder Not Otherwise Specified; NG: Nasogastric; OTA : Occupational Therapy Assistant; BMI: Body Mass Index; RED: Restrictive Eating Disorder

Study designs included three retrospective chart studies [12, 27, 29], a longitudinal study [26], a cross-sectional study [28] and a randomised pilot study [25].

### Intervention characteristics

Assistance provided at mealtimes included direct supervision by qualified (e.g., trained healthcare professional) or unqualified (e.g., parents) subjects. However, no study compared the difference between these two types of intervention and the relative implication in the mealtime assistance. In addition, the subject in charge of meal supervision sat with the patient, or group of patients, throughout either the meal or snack, providing empathy and understanding of the patient’s struggle, setting firm expectations about what had to be eaten in a set period of time [29], providing interpersonal support and conversation during the meal [12, 26], encouraging food consumption [12], and avoiding topics of body weight, body image, and calories from food items and beverages [27].

A summary of key areas of investigation of the selected studies and their respective findings are summarized in Table 3. Studies explored the role of assisted meal in improving body weight [12, 25, 27], vital signs [12, 27], the duration of hospitalization and the rate of readmission to hospital [12, 27, 29]. Two studies considered the experience, the feelings and the psychological effects of mealtime supervision (assessed through questionnaires and interviews) experienced by both patients and staff members involved compared to a fully independent management of the meals [26, 28]. One study assessed changes in eating behaviour and in mealtimes length after a treatment of meal supervision [26]. One study evaluated, in addition to the duration of hospital stay, the risk of readmission, body weight recovery rate and the reduction in the incidence of the use of the Nasogastric (NG) tube in patients hospitalized under meal supervision [29].

### Nutritional outcomes

Mealtime assistance in hospitalised patients with a diagnosis of AN reduced the need of NG feeding (67% versus 11%, *p* < 0.02) [29]. However, no differences were observed in length of stay, respectively, in hospitalised patients with AN receiving meal support therapy (*p* < 0.39) [29] and in hospitalised patients in pre-diagnosis, with a diagnosis of Eating Disorder Not Otherwise Specified (EDNOS) or AN who received meal supervision from admission (*p* = 0.27) [27]. Coutrier et al. reported no differences in the readmission rate in hospitalised patients with a diagnosis of AN (*p* < 0.66) [29].

### Anthropometric outcomes

Results from a randomized study showed that assisted meals do not have a significant effect on weight recovery in outpatients with a diagnosis of AN (CI 95%, *p* = 0.39) [25]. In addition, in a retrospective chart study on supervised patients hospitalized with Restrictive Eating Disorders (RED) exceeded the weight gain goal of 0.2 kg/day (mean gain of 0.35 kg/day vs. 0.33 kg/day in those who did not receive meal supervision, *p* = 0.52) [12]. Another study by Kells et al. [27] reported no significant differences in weight gain per day (*p* = 0.63) and in in the mean percentage of days that goal weight gain was achieved (*p* = 0.78) for those who received meal supervision from admission.

Nevertheless, evidence from an observational study demonstrated that the mean BMI significantly increased during the hospital stay in patients with AN or BN under direct meal supervision, (15.3 kg/m^2^ at admission to 16.0 kg/m^2^ at discharge, *p* = 0.001) [28]. Moreover, a higher rate of weight gain (kg/week) was showed in hospitalised patients with a diagnosis of AN receiving meal support therapy (*p* < 0.09), even if the change in body weight was not significant (*p* < 0.17) [29].

### Psychological outcomes

Only one study has described the psychological experience of parents with EDs. Parents of hospitalised patients with AN or BN were especially pleased to have meal planning taken over by dietitians and reported benefit from supervision of meals at home on a five-point Likert scales ranging from 1 = very unhelpful to 5 = very helpful (4.34 points vs. 2.82 points, *p* = 0.001) [28]. Conversely, the psychological perspective of the patients themselves has not been extensively explored or taken into account.

### Behavioural outcomes

Qualitative results from the only study that explored the behavioural changes showed that mealtime assistance for inpatient AN patients reduced the average number of ED behaviours per patient by 35% and reduced distress and anxiety for patients and staff. In addition, the average length of mealtimes across a week reduced from 53 to 43 min [26].

### General health and medical complications

Qualitative analysis from a pilot study reported a normalisation in the menstrual function in 5/6 outpatients with a diagnosis of AN receiving meal assistance (vs. 2/6 of the control group) [25]. No significant differences in serum electrolytes or vital signs (e.g., low heart rate, *p* = 0.73) were reported in hospitalised patients with ED NOS or AN receiving mealtime supervision [27] and supervised patients with RED reported fewer episodes of overnight bradycardia, but still not statistically relevant (15% vs. 40% than patients not receiving meal supervision, *p* = 0.20) [12].

### Data extraction and quality assessment

Extraction parameters centred on study characteristics (such as author, year, setting, intervention and comparator details, data collection duration, level of evidence and population characteristics) and outcomes (including nutritional, anthropometric and eating behavioural outcomes, psychological aspects). Tables 4 and 5 summarise the results of the study quality assessment. The quality of the studies was assessed using the Newcastle Ottawa Scale, with a version appropriately modified for cross-sectional studies assessment [30], and with Version 2 of the Cochrane risk-of-bias assessment tool [31] for the only randomised trial included [25]. The average score obtained from the application of the Newcastle Ottawa Scale (NOS) for non-randomised studies was 4.5/10 (range: 3–6/10). For the evaluation of the randomised study [25], two authors (DL and EM) independently examined the study of interest for the following sources of bias: randomisation process, deviations from intended interventions, missing outcome data, measurement of the outcome, selection of the reported result and overall bias. The methodological quality assessments of pilot study showed an inappropriate procedure for a randomly generated sequence and the absence of information about allocation and blinding. The study is deemed to have substantial concerns across multiple domains, thereby significantly reducing confidence in the results.

**Table 4:** NOS for quality assessment of non-randomized studies [30]

	Selection (0–5 stars)	Comparability (0–2 stars)	Outcome (0–3 stars)	Total score
Bravender et al. (2017) [28]	2	0	1	3
Coutrier et al. (2009) [29]	3	0	2	5
Gardner et al. (2021) [26]	2	0	1	3
Kells et al. (2013) [12]	3	0	2	5
Kells et al. (2017) [27]	3	0	3	6

**Table 5:** Version 2 of the Cochrane risk-of-bias assessment tool for randomised trials [31]

	Bias arising from the randomisation process	Bias due to deviations from intended interventions	Bias due to missing outcome data	Bias in measurement of the outcome	Bias in selection of the reported result	Overall bias
Herscovici et al. (2006) [25]	Some concerns	High	High	High	Some concerns	Some concerns

Low, some concerns and high indicate risk of bias

## Summary of the data

The synthesis of the study results was achieved through discussion and in a narrative–descriptive form. A quantitative synthesis (meta-analysis) has not been carried out because of the heterogeneity in the definition of meal assistance intervention, in the modalities of conducting assisted meal, in the figures involved in meal assistance, in the different types of patients considered in the sample (age, type and severity of EDs), in the various treatment settings and in the different outcomes considered in the various studies selected in the systematic review.

## Discussion

This systematic review summarises existing evidence assessing the effectiveness of assisted meals on nutritional, anthropometric, psychological and behavioural outcomes in patients with EDs.

Despite the marked heterogeneity in the definition and methodological approaches of assisted meal and the consequent and concomitant absence of a significant number of clinical studies that measure its therapeutic efficacy in the context of EDs, the primary finding highlights preliminary evidence of the positive impact that meal assistance could have on the rehabilitation treatment of EDs.

In spite of the increasing attention and awareness by clinicians about the relevance of meal assistance in a multidisciplinary treatment within the care settings for EDs, research on this therapeutic procedure is scarce, especially due to a lack of systematic, standardised approaches. The causes of this observation can be traced in the different approaches to the treatment of ED. The extreme variability of definition and methodology can be attributed, at least partially, to the different settings where studies were performed and to the variability in specialties and backgrounds of the researchers. Furthermore, it is noteworthy that assisted meals in clinical practice are predominantly employed to support weight restoration in patients with restrictive eating behaviours.

The general results of this systematic review show overall evidence of improvement in patients suffering from EDs and undergoing meal care.

In terms of nutritional status, evidence remains limited and somewhat contradictory. Kells et al. reported a shorter length of stay (by 3 days) for those receiving consistent meal supervision from admission, compared to those who received meal supervision “as needed”, for instance, when patients did not meet weight gain targets or could not complete meals [27]. This finding contrasts with another study in which the length of time spent in hospital, as well as the readmission rate, did not change considerably in favour of patients undergoing meal support therapy [29]. However, meal assistance was associated with a substantial reduction, from 67% to 11%, in the use of NG tubes [29]. None of the assessed results were statistically significant in the quantitative analysis. These inconsistencies and apparent lack of effects on nutritional status might be attributed to the study designs. Preliminary beneficial changes were observed, underscoring the vast research gap in this area and the pressing need for further investigation. Regarding anthropometric parameters, data are mixed. In two studies there were no significant changes, in terms of body weight regain, in patients subjected to the program with assisted meal [27, 29]. However, another study showed that in hospitalized patients, supervision during meals provided benefits in terms of body weight restoration, but not with consistent clinical significance [12]. Limited evidence from anthropometric data creates excellent opportunities for future research to further understanding of the role of mealtime assistance in patients with EDs.

The psychological outcomes mainly describe the point of view of parents and relatives. The experience of the assisted meal was reported positively, not only by the patients directly involved in the program, but also by the families of teenagers affected by EDs. Although the possible bias due to the small sample size and the evaluation of psychological aspects using scales and open-ended questions, parents and patients particularly appreciated staff supervision of meals and they were especially supportive of having meal planning taken over by a dietitian, compared to an autonomous home management [28]. However, limited research has addressed the impact that the presence of parents or family members could potentially have on the effects of meal assisted therapy and no study assessed these outcomes in patients with EDs.

Evidence from one study highlighted behavioral outcomes showing that assisted meals have beneficial effects especially in patients with severe psychopathology of eating disorders [25]. In addition, results from another study indicated a reduction in dysfunctional eating behaviour, such as the avoidance of conviviality during meals. Nonetheless, the absence of statistical analysis does not consent to draw any consistent conclusion on this outcome [26].

Focusing on medical complications, although supervised patients reported fewer episodes of nocturnal bradycardia [12], the rest of the vital signs were not significantly improved for those receiving assistance during the meal [27]. To gain a deeper understanding of the effects of mealtime assistance on the vital signs, it would be beneficial to delve deeper with further well-designed studies that have larger sample sizes.

None of the studies in the review specifically considered potential negative effects of mealtime assistance as outcomes.

Nevertheless, no negative effects of the procedure have been reported by the studies analysed.

Furthermore, on the available evidence, no difference could be concluded regarding a more beneficial effect of the mealtime assistance provided by healthcare staff, volunteers, or family members.

The qualitative analysis indicated a low overall quality, with all NOS scores falling below 7. In addition, the judgment of the sole study assessed using the "Cochrane Risk of Bias Tool" was rated as having "some concerns" in terms of overall risk bias. Low quality and the lack of univocity in the evidence is a limiting factor to draw unambiguous conclusions about the therapeutic role of the assisted meal. Improving quality of study designs would allow a better assessment of the effectiveness of meal assistance and improve the reliability and validity of future research on the topic.

### What is already known on this subject?

It is well-established that the dining room has been identified as a difficult area for patients with EDs that experience elevated level of distress and anxiety and exhibit a high number of ED behaviours, which act as maintaining factors for the disorder [26]. In addition, especially RED patients, experience change in brain activity in response to food stimuli and behavioural rigidity that make mealtimes peculiarly difficult [32]. Mealtime assistance could be part of a therapeutic intervention to enhance cognitive behaviour through the development of skills and strategies, to address the common maintaining factors for patients, enabling have a potential positive impact on the psychopathology of the illness. Despite that, there is scarce evidence on the impact of the meal assistance on patient care as well as a lack of resources and clarity on how to best support during meals [22, 26, 29].

The main research into mealtime assistance in hospitalised patients explored the perspectives of patients with AN [20]; patient satisfaction surveys highlighted meal support therapy as one of the most helpful aspects of the treatment of inpatients with EDs [22]. The authors focused on how the care staff manage patients’ mealtimes; for example, whether rules should be adhered to [33], how to approach with a patient during mealtimes, and described their practical strategies for supporting patients during mealtime, such as role modelling [34].

Adapting the approach of mealtime assistance in the community settings has been identified as potentially beneficial, despite the effectiveness of the intervention is poorly evidenced in community [20].

### What does this study add?

In a context of limited evidence, our findings suggest that supervision of meals may have a role in addressing disordered behaviour and encouraging healthy eating habits.

Based on our review, a specific need emerged for formal and standardised method to meal assistance approach with a view to guarantee an adequate meal management and have a potentially positive impact in clinical practice.

The present findings will hopefully provide preliminary evidence for future researchers and worksite policy to take into consideration mealtime management as a valuable intervention. Besides, our observations may increase public perception of the importance of supporting patients with EDs during mealtime in various settings.

## Strengths and limits

To the best of our knowledge, the current review is the first one to develop an aggregated synthesis of qualitative data on assistance at mealtimes for patients with ED. Indeed, a point of strength is that it has examined not only the impact of mealtime assistance on clinical outcomes, but also the patients, and staff perspective and experience during the meal situation. In addition, we found it striking to include and compare studies that investigated the structure of mealtimes, the mealtime activities, the interactions and the patients’ involvement, to enhance awareness in future interventions to support patients during mealtimes.

The limitations of the systematic review mainly concern a considerable heterogeneity among the selected studies, which includes variability in study designs, sample sizes, data collection, and outcomes. A strong heterogeneity has been found in the execution of the intervention and the kind of patients included. In organizational contexts of the assisted meal program, it would be useful to adopt standardised outcome measures, both for the specific disorder and for the physical comorbidities, to monitor the effectiveness of treatments and the impact on specific conditions. A clear definition of how the assisted meal is carried out and the use of objective measures to assess the improvement of anxiety symptoms and dysfunctional behaviours, would significantly reduce the risk of bias and improve the ability to compare results. The use of a standardized approach to treatment outcomes would facilitate the comparison of different interventions and optimize the actions to be carried out in the therapeutic–rehabilitation programs in patients with EDs.

Further studies with a stringent methodology are needed to assess the actual sensitivity, specificity and validity of the assisted meal by applying the assisted meal procedures to large cohorts of patients.

## Conclusions

To our knowledge this is the first systematic review to examine the role of assisted meal in patients with EDs. Despite the methodology of meal supervision in patients with EDs is not thoroughly delineated, available evidence suggests that the assisted meal management model, provided by qualified healthcare staff, volunteers or family members, might be helpful in improving nutritional–rehabilitation treatment in patients with EDs. Within the different therapeutic setting (e.g., community-based care, inpatient and outpatient care), assisted meal intervention is carried out according to specific individual or group work and it is related to an overall improvement of dysfunctional eating behaviour (e.g., dietary restriction, binge eating, compensation behaviour) and seems to be a logical intervention to foster healthy eating habits.

Meal assistance can provide necessary support, distraction, and decentralization from concerning factors that obstruct meal intake, contribute to the to the patients’ understanding of the disorder and its manifestations and consequences, thus educating patients on the process of adopting a normal eating pattern. Contrasting evidence remains from the reduction in hospitalization length and anthropometric parameters that did not significantly improve in patients with EDs undergoing meal assistance program.

The results do not allow definitive conclusions to be drawn from a clinical and functional perspective. Despite an overall improvement in general state of EDs patient, a firm recommendation for the implementation of mealtime assistance programs in patients with EDs cannot yet be made given the limited research to date because of the scarce number and great heterogeneity of included papers. Increased focus on meal management and the sequence of instructions to be followed could help clinical staff to improve awareness about the right approach to be adopted for meal assistance during the various phases of the disease, and to allow mealtime assistance programs to be implemented and better structured. Additional research is necessary to clarify whether the assisted meals can be an effective mean of addressing, systematically, the treatment, i.e., the nutritional rehabilitation of patients with EDs.

### Relevance to clinical practice/next steps and directions for future research

The research shows that a coordinated and integrated multidisciplinary management between healthcare professionals and the involvement of family members, can be beneficial in patients with EDs. Our findings indicate that mealtime management by experienced clinicians may positively influence treatment. There is a requirement to develop evidence-based supervision programmes that could be widely operationalized. Additional studies should be carried out to measure efficacy [21]. In the future, a rules-based approach, when delivering the mealtime management intervention, could help breaking down entrenched eating disordered behaviours [16]. Future guideline development should also be based on perspectives from individuals who have experienced eating disorders and participated in successful mealtime management approaches [20].

## Data Availability

The data that support the findings of this study are available from the corresponding author, EM, upon reasonable request.

## Abbreviations

AN: Anorexia Nervosa
BED: Binge Eating Disorders
BN: Bulimia Nervosa
EDNOS: Eating Disorder Not Otherwise Specified
EDs: Eating disorders
NG: Nasogastric
NOS: Newcastle Ottawa Scale
OTA: Occupational Therapy Assistant
PICO: Population Intervention Comparator Outcome
PRISMA: Preferred Reporting Items for Systematic Reviews and Meta-Analyses
RED: Restrictive Eating Disorder
SR: Systematic review
